# Herpesviruses mimic zygotic genome activation to promote viral replication

**DOI:** 10.21203/rs.3.rs-3125635/v1

**Published:** 2023-12-13

**Authors:** Florian Full, Stephanie Walter, Eva Neugebauer, Jiang Tan, Nir Drayman, Vedran Franke, Savaş Tay, Markus Landthaler, Altuna Akalin, Armin Ensser, Emanuel Wyler

**Affiliations:** University Medical Center, and Faculty of Medicine, Albert-Ludwig-University Freiburg; Institute for Clinical and Molecular Virology, University Hospital Erlangen; Institute of Virology, University Medical Center, and Faculty of Medicine, Albert-Ludwig-University Freiburg; Institute of Virology, University Medical Center, and Faculty of Medicine, Albert-Ludwig-University Freiburg; The Department of Molecular Biology and Biochemistry, the Center for Virus Research and the Center for Complex Biological Systems, The University of California, Irvine; Max Delbrück Center for Molecular Medicine; University of Chicago; Max-Delbrueck-Center; Max Delbrueck Center; University Hospital Erlangen; Max-Delbrueck-Center

**Keywords:** Herpesvirus, DUX4, zygotic genome activation, FSHD, endogenous retroelements, HSV-1, HSV-2, HCMV, EBV, KSHV, HPV

## Abstract

DUX4 is a germline transcription factor and a master regulator of zygotic genome activation. During early embryogenesis, DUX4 is crucial for maternal to zygotic transition at the 2–8-cell stage in order to overcome silencing of genes and enable transcription from the zygotic genome. In adult somatic cells, DUX4 expression is silenced and its activation in adult muscle cells causes the genetic disorder Facioscapulohumeral Muscular Dystrophy (FSHD).

Here we show that herpesviruses from alpha-, beta- and gamma-herpesvirus subfamilies as well as papillomaviruses actively induce DUX4 expression to promote viral transcription and replication. We demonstrate that HSV-1 immediate early proteins directly induce expression of DUX4 and its target genes including endogenous retroelements, which mimics zygotic genome activation. We further show that DUX4 directly binds to the viral genome and promotes viral transcription. DUX4 is functionally required for herpesvirus infection, since genetic depletion of DUX4 by CRISPR/Cas9 abrogates viral replication.

Our results show that herpesviruses induce DUX4 expression and its downstream germline-specific genes and retroelements, thus mimicking an early embryonic-like transcriptional program that prevents epigenetic silencing of the viral genome and facilitates herpesviral gene expression.

## Introduction

Herpesviruses are a major health burden worldwide, with a prevalence of 25 – 100% dependent on the virus species and geography ^1,2^. Herpesviruses cause a number of prevalent diseases, like oral and genital herpes, chickenpox, shingles, infectious mononucleosis and encephalitis. Life-threatening infections in healthy humans are rare, however severe disease is a significant risk in e.g. immunocompromised patients, transplant recipients or newborns ^3,4^. Some herpesviruses such as Epstein-Barr virus (EBV) and Kaposi’s sarcoma-associated herpesvirus (KSHV) are also known to be the causative agent of particular cancer types ^5^. Due to their lifelong persistence in the host, the risk of reactivation and developing a lytic infection is constantly present. Treatment options are limited and therapies able to eliminate the virus from the body are unavailable. A better understanding of the mechanisms involved during herpesviral infection and persistence is therefore essential.

In a recent study, we observed that the upregulation of the cellular protein Tripartite-motif 43 (TRIM43) upon HSV-1 infection is dependent on the embryonic transcription factor DUX4 ^6^, which was also confirmed by Friedel et al. ^7^. DUX4 is a germline transcription factor exclusively expressed during the 2-cell to the 8-cell state of embryogenesis, a short phase during human embryonic development ^8–10^. There, DUX4 is critical for zygotic genome activation (ZGA), a transcriptional activation event that leads to the induction of hundreds of target genes and allows the embryo to proceed further in development ^11,12^. Concomitantly, it induces retroelements such as LTRs and ERVs ^9,10,13^ which function as alternative promotors, regulate ZGA-related genes and mediate pluripotency ^14,15^. After this limited period of activation, DUX4 is silenced throughout all adult tissues, except for spermatocytes ^16^. An exception to this is the genetic disorder Facioscapulohumeral Muscular Dystrophy (FSHD). In FSHD, DUX4 silencing is lost and aberrant DUX4 expression in adult muscle cells leads to apoptosis and degeneration of the affected muscles ^17,18^. The DUX4 gene resides within the macrosatellite repeat array D4Z4 ^19^, that harbors up to 100 copies of DUX4, with only the most distal copy encoding for a protein ^17^. During early embryonic development, this macrosatellite array is transformed into heterochromatin after the 8-cell stage ^8,10^ and its repression is strictly controlled by chromatin remodeling factors and repressors which have not been fully investigated ^16,19^.

Given the peculiar expression pattern of DUX4 and its upregulation in herpesviral infection, we aimed to determine whether DUX4 plays a role during herpesviral infection and to shed light on its potential functions. We show that DUX4 expression during herpesviral replication is a general mechanism that is conserved among herpesviral subfamilies and can also be observed in patient samples. For HSV-1, we could demonstrate that the viral proteins ICP0 and ICP4 induce DUX4 expression and hundreds of DUX4 target genes, including several classes of endogenous retroelements, and that DUX4 is essential for HSV-1 and HSV-2 replication. This points at so far unknown endogenous functions of DUX4 and extends our knowledge of this unique transcription factor.

## Results

**Figure 1:**
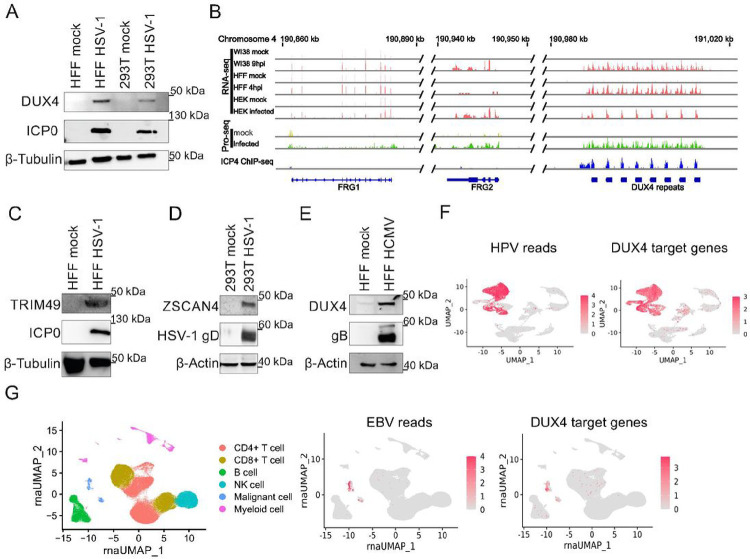
**A** Western blot of primary HFF cells infected with HSV-1 for 24 h and 293T cells infected with HSV-1 for 18 h, both MOI 5. ICP0 was used as marker for infection. **B** RNA-Seq. data of the DUX4 and neighbouring FRG1 and FRG2 loci in WI38 9 hpi, HFF 4 hpi and HEK 293T cells 18 hpi. The Precision Run-On Sequencing (Pro-Seq.) data show RNA POL II occupancy on the FRG1, FFRG2 and DUX4 locus in infected and uninfected cells. ICP4 ChIP-Seq. data show ICP4 occupation at the DUX4 locus in cells infected with HSV-1. Western blot of HFF cells infected with HSV-1 for 24 h with MOI 5. **C** Western blot of HFF cells infected with HSV-1 for 18 h with MOI 5 and analyzed for expression of TRIM49. ICP0 served as a control for viral infection. **D** Western blot of 293T cells infected with HSV-1 for 18 h with MOI 5 and analyzed for expression of ZSCAN4. Glycoprotein D (gD) served as a control for viral infection. **E** HFF cells infected with HCMV for 6d with MOI 1. Western blot analysis of DUX4. HCMV glycoprotein B (gB) was used as marker for infection. **F** Reanalysis of single cell sequencing datasets from patients with HPV-pos. head and neck cancer from Kürten et al. ^42^. Left panel: UMAP projection of number of HPV-specific reads per cell, pool of 10 donors, 5 donors HPV positive and 5 donors HPV negative. Right panel: UMAP projection of number of DUX4-target gene specific reads, pool of 10 donors, 5 donors HPV positive and 5 donors HPV negative.**G** Reanalysis of single cell sequencing datasets from patients with EBV-pos. Nasopharynx carcinoma (NPC) from Liu et al.^43^. Left panel: cell identity map. Middle panel: UMAP projection of number of EBV-specific reads per cell, pool of 10 donors. Right panel: UMAP projection of number of DUX4-target gene specific reads, pool of 10 donors.

We confirmed the expression of DUX4 in various cell lines infected with HSV-1 by Western Blot and reanalysis of published RNA-seq. datasets (Fig. 1A–B) ^6,20,21^. Comparison of three independent RNA-seq datasets in 3 different cell types showed no expression of DUX4 and DUX4 target genes (e.g. FRG1) in uninfected cells (Fig 1B). Upon infection with HSV-1, however, transcription is induced for DUX4 as well as known DUX4 target genes (Fig. 1B). Interestingly, we also confirmed the expression of HSAT-II satellite repeat transcripts. HSAT-II repeats are known DUX4 targets ^10,22^ and have recently been shown to play a role in HCMV replication ^23^ (Fig. S1a). Moreover, the reanalysis of a published Precision Run-On sequencing (PRO-seq.) dataset ^24^ showed no RNA-polymerase-II occupancy at the DUX4 locus in uninfected cells, indicating that the locus is completely silenced under normal conditions (Fig. 1B). In contrast, HSV-1 infection induced a robust PRO-seq signal at the DUX4 locus. DUX4 expression was also induced upon infection of primary human melanocytes with HSV-1, emphasizing the physiological relevance of DUX4 induction (Fig. S1b). Western Blot as well as qRT-PCR experiments further confirmed the expression of DUX4 and known DUX4-target genes TRIM48, TRIM49, ZSCAN4, ZSCAN5a, ZSCAN5d and RFPL4A upon HSV-1, HCMV and KSHV-infection (Fig. 1C–E, Fig. S2A-G) ^25^ in different experimental settings. This indicates that DUX4 is functional and acts as a transcriptional activator in herpesvirus infected cells. Reanalysis of published RNA-seq. datasets could not confirm expression of DUX4 in cells infected with other RNA-viruses or DNA-viruses like Adenoviruses or Poxviruses (Fig. S3A and data not shown). However, we could detect expression of DUX4 and DUX4 target genes in single cell RNA-seq. datasets from human Papillomavirus (HPV) positive head and neck cancer patients (Fig. 1F). By reanalyzing single cell RNA-Seq.- data from patients with EBV-pos. nasopharyngeal carcinoma, we could further demonstrate that DUX4 target gene expression was only detected in EBV-pos. tumor cells but not in healthy tissue from the same patients (Fig. 1G). Taken together, these data show that the induction of DUX4 expression is a common feature of human herpesviruses and papillomaviruses and of relevance *in vivo*.

**Figure 2:**
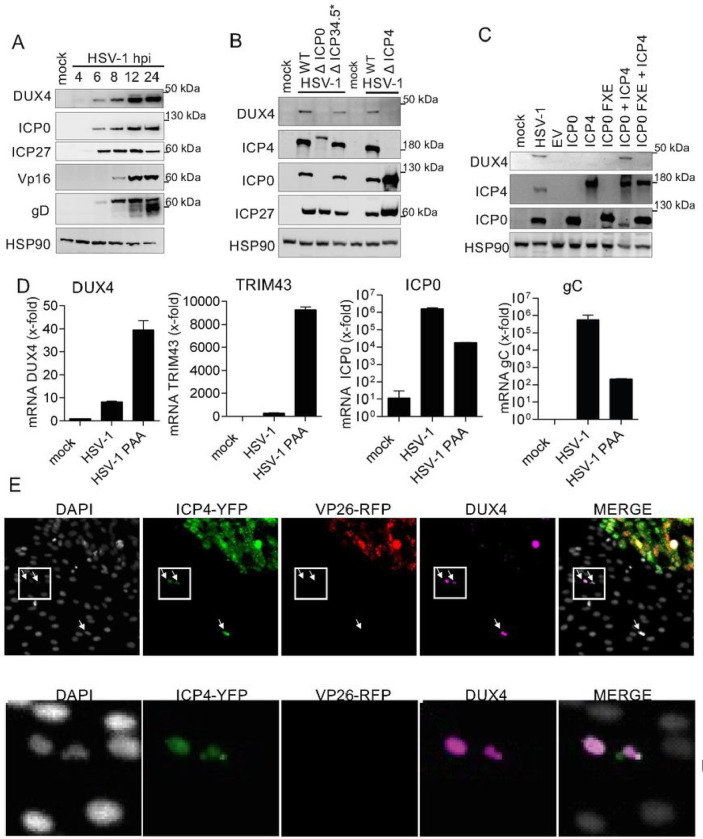
Herpesviral immediate-early proteins induce DUX4 expression **A** Western Blot of DUX4 and HSV-1 proteins ICP0, ICP27, VP16 and glycoprotein D (gD) in HFF cells infected with HSV-1 harvested at different hpi (MOI 10). **B** Western Blot of DUX4 and HSV-1 proteins in primary HFF infected with HSV-1 and HSV-1 mutants harvested at 16 hpi (MOI 10). One representative experiment out of n=5. **C** Western blot analysis of DUX4 and HSV-1 protein in 293T cells transfected with HSV-1 IE proteins ICP0, ICP0 FXE, ICP4 (ICP4-YFP), ICP0 + ICP4 (ICP4-YFP) and ICP0 FXE + ICP4 (ICP4-YFP) for 48 h or infected with HSV-1 for 18 h (MOI 10). ICP0 FXE is a mutant with a deletion in the RING domain, which inhibits Ubiquitin E3 ligase activity. EV: empty vector control. One representative experiment out of n=3 **D** qRT-PCR analysis of cellular genes DUX4, TRIM43 as well as the viral genes ICP0 and UL36 in HDF-TERT cells untreated or treated with PAA and infected with HSV-1 (MOI 0.1). Values are presented as fold induction (normalized to HPRT RNA) relative to uninfected control cells. **E** Immunofluorescence of HDF-TERT cells infected with a recombinant HSV-1 expressing ICP4-YFP and VP26-RFP. DUX4 protein was stained with a DUX4 specific antibody, nuclei were counterstained with DAPI.

Next, we wanted to address the significance of herpesviral DUX4 induction for the infection itself, since DUX4 is a germline transcription factor that is not expressed in healthy adult tissue ^8,10^. We first hypothesized that the induction of DUX4 might be part of an antiviral response of the cell to viral infection triggered by a herpesviral pathogen associated molecular pattern (PAMP), or by herpesviral induction of a cellular DNA-damage response (DDR). We reanalyzed several published RNA-sequencing datasets, investigating DUX4 mRNA expression in response to cellular changes, but found no evidence for DUX4 expression (data not shown). In addition, triggering a DNA-damage response by treating cells with Bleocin or Etoposide did not induce DUX4 expression (Fig. S3A). The only evidence for DUX4 expression came from a Chromatin-IP-sequencing (ChIP-Seq.) dataset ^26^ investigating the binding of the HSV-1 infected cell protein 4 (ICP4) to the cellular genome. ICP4 is a potent activator of viral transcription, part of the viral tegument, and essential for replication of HSV-1^27,28^. Our reanalysis of this dataset showed a very strong binding of the ICP4 protein to the DUX4 locus (Figure 1B). Of note, the peak intensity of ICP4 binding at the DUX4 locus was among the highest in the entire host genome. This ICP4 binding was a strong indication for an active induction of DUX4 by HSV-1 infection. Moreover, experiments with phosphonoacetic acid (PAA), an inhibitor of the viral DNA-polymerase, showed that herpesviral DNA replication is dispensable for DUX4 expression (Fig. S3B), indicating that induction of the DUX4 gene takes place at the immediate-early stage of the viral gene expression cascade. Even after infection with UV-inactivated virus, DUX4 protein was still induced, indicating that incoming components of the virion contribute to DUX4 induction (Fig. S3B). Analysis of the kinetics of DUX4 expression upon HSV-1 infection further demonstrated that DUX4 protein could be detected as early as 4h post infection with protein levels constantly increasing over the course of infection (Fig. 2A). Reanalysis of RNA-sequencing data from Rutkowski et al.^21^ further demonstrated that known DUX4 target genes have variable dynamics during infection with most DUX4 target genes being upregulated at 3–4 hours post infection (Fig. S4). To elucidate the involvement of HSV-1 tegument proteins in the induction of DUX4 expression we performed infection experiments with HSV-1 mutants depleted of the immediate early proteins ICP0, ICP4 and gamma-34.5. We could show that wildtype (wt) HSV-1 infection as well as infection with HSV-1-Δ γ34.5 resulted in DUX4 expression, whereas DUX4 expression is abrogated in cells infected with HSV-1 lacking either ICP0 or ICP4 (Fig. 2B and Fig. S5A). In addition, the transient co-expression of ICP0 and ICP4 is sufficient to induce DUX4 expression in the absence of viral infection (Fig. 2C), whereas an E3-ligase deficient mutant of ICP0 (ICP0 FXE) was not able to induce expression of DUX4 when coexpressed together with ICP4. This confirms that ICP0 and ICP4 are necessary and sufficient for inducing DUX4 expression.

DUX4 is a transcription factor active in the nucleus where it executes its physiological function by activating its target genes. We used a recombinant HSV-1 expressing ICP4-YFP and VP26-RFP in order to visualize progression of infection and localization of DUX4. ICP4 is an immediate-early gene and part of the tegument, whereas VP26, the small capsid protein of the virus, is expressed late in the infection cycle. By co-staining of cells with a DUX4 antibody, we could demonstrate that only ICP4+/VP26− cells (YFP+/RFP− cells) show nuclear expression of DUX4, indicating activation of DUX4 within early stages of infection (Figure 2E). In contrast, ICP4+ and VP26+ cells (YFP+ and RFP+ cells) show either no DUX4 expression or an aberrant cytoplasmic localization of DUX4. This indicates that DUX4 is only briefly activated by ICP0 and ICP4 during the early phase of infection. To test this, we treated cells with PAA, which arrests viral infection at the stage of viral DNA replication, i.e. after immediate-early gene expression but before late gene expression (Figure 2D and Fig. S5B). PAA treated cells showed a strong increase in expression of DUX4 and its target genes by qRT-PCR analysis of HDF cells infected with HSV-1 (Figure 2D), as well as the DUX4 protein by WB analysis in 293T cells (Figure S3B), supporting the notion of a transient activation of DUX4 during the early stages of HSV-1 infection.

**Figure 3:**
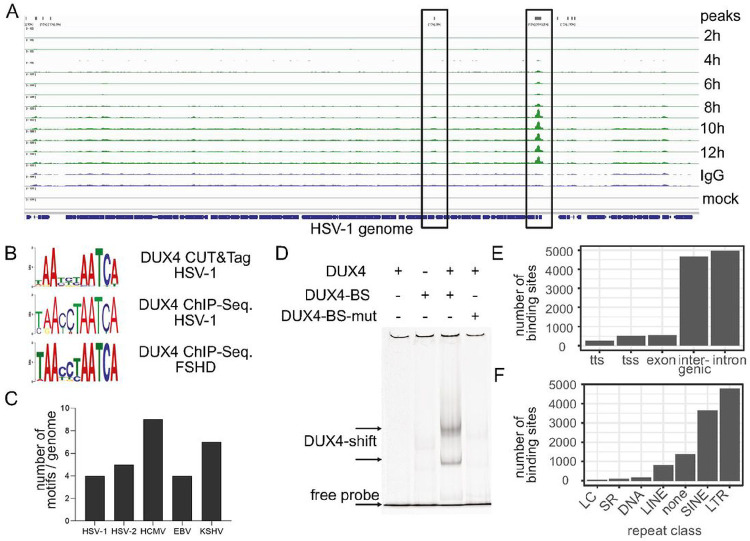
Direct binding of DUX4 to the HSV-1 genome **A**CUT&Tag with a DUX4-specific antibody in HFF cells infected with HSV-1 at 8 hpi and 14 hpi. **B** Comparison of DUX4 consensus binding sequence from DUX4 CUT&Tag in HSV-1 infected cells (upper panel), DUX4 HSV-1 ChIP-Seq. (middle panel) and with the previously published DUX4 consensus sequences (Geng et al.) in the lower panel. **C** Number of DUX4 consensus binding site found in the genomes of HSV-1, HSV-2, HCMV, EBV and KSHV. **D** Electrophoretic Mobility Shift Assay (EMSA) of 600bp fragments of fluorescently labelled viral DNA containing one DUX4 binding motif (DUX4-BS), incubated with purified DUX4 protein (DUX4). The mutated binding site (DUX4-BS-mut) contains a T->C nucleotide exchange at position 9 of the binding motif. **E** Number of DUX4 binding sites in different regions of the human genome. (TTS: transcription termination site site, TSS: transcription start site) **F** Analysis of DUX4 binding sites that can be found within repetitive elements. (LC: low complexity repeats, SR: simple repeat)

**Figure 4:**
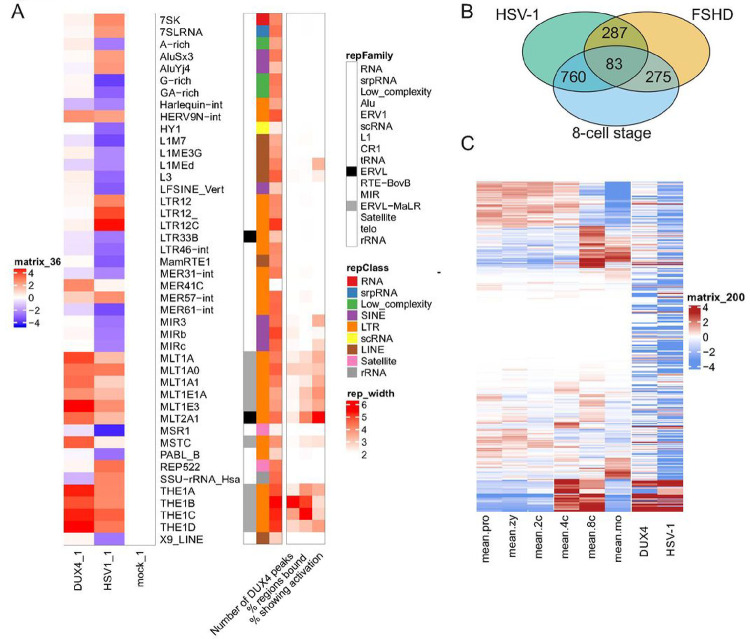
HSV-1 infection results in activation of endogenous retroelements. **A** Repetitive elements regulated in DUX4 overexpressing cells compared to HSV-1 infected cells reanalysed from Full et al. 2019 ^6^ and combined with our ChIP-Seq data. On the right site repetitive elements are ascribed to their respective class and family. DUX4 binding is scaled to repeat size, number of peaks and activation. **B** Venn diagram showing overlap of genes that are significantly upregulated in the 8-cell stage of human development, HSV-1 infection and FSHD. **C** Expression pattern of embryonic genes during embryonic genome activation compared to expression pattern in DUX4 overexpressing and HSV-1 infected cells. Embryonic data is normalized to the average expression value of each gene. Data from DUX4 overexpression and HSV-1 infection is relative to mock. Shown are genes which have DUX4 binding sites in the proximity of the TSS. (pro: pronucelus, zy: zygote, 2c: 2-cell state, 4c: 4-cell state, 8c: 8-cell state, mo: morula)

It is known that the physiological function of DUX4 during embryonic genome activation at the 2–8 cell stage is to directly bind to DNA and activate genes and retroelements that are necessary for developmental progression ^9,10^. In particular endogenous retroelements act as promoters for downstream genes, and binding of DUX4 to retroelements activates transcription of respective genes ^14,29^. In order to analyze the role of DUX4 in herpesviral replication, we performed endogenous DUX4 ChIP-seq. as well as DUX4 CUT&Tag experiments. The DUX4-ChIP conditions optimized for DUX4 binding to the cellular genome resulted in a high background for the viral genome, most likely due to different physical properties that affect sonication and differences in chromatin accessibility. However, using CUT&Tag we could detect direct binding of DUX4 to the HSV-1 genome at about 4h post infection (Fig. 3A). We observed several DUX4 binding sites over the HSV-1 genome, with the biggest peak located in between the UL55 and UL56 genes and increased binding over time. Analysis of the binding sites from our endogenous DUX4 ChIP-Seq, CUT&Tag and comparison with the DUX4 ChIP-Seq performed by Young et al.^14^ with overexpressed DUX4 protein in muscle cells showed almost identical consensus binding sites (Fig. 3B). A computational analysis found between 4–9 potential DUX4 consensus motifs in the genomes of HSV-1, HSV-2, HCMV, EBV and KSHV (Fig. 3C), suggesting that DUX4 possibly binds to viral genomes of all herpesviruses. Next, we established an electrophoretic mobility shift assay to confirm binding of DUX4 to regions of the HSV-1 genome (Fig. 3D). To this end, full-length DUX4 protein was expressed in E.coli (Fig. S6), purified and about 600bp-long fragments of the HSV-1 genome amplified by PCR. After coincubation of recombinant DUX4 with fluorescently labeled PCR fragments we observed binding of DUX4 to a fragment containing one copy of the consensus DUX4 DNA-binding motif, whereas the exchange of T->C at position 9 of the binding motif completely abrogated binding (Fig. 3D). For the host genome, the ChIP-Seq and the CUT&Tag experiments revealed about 11000 DUX4 binding sites for ChIP-Seq and 3700 DUX4 binding sites for CUT&Tag within the host genome. Surprisingly, most DUX4 binding sites were not at transcriptional start sites or within exon regions of genes, but within intronic and intergenic regions (Fig. 3E). Analysis of intronic / intergenic binding sites showed a strong preference of DUX4 binding to repetitive genetic elements (Fig 3F). We found predominant binding of DUX4 to long-terminal repeat (LTR-) elements and short interspersed nuclear elements (SINE) and only a small fraction of binding sites in actual genes (Fig. 3F). During early embryonic development and in particular ZGA, it is known that activation of endogenous retroelements can generate alternative promoters for expression of genes, resulting in transcription of developmental genes that are essential for further embryonic development ^15,30^. Analysis of our RNA-seq and DUX4 ChIP-seq. data sets showed a DUX4-mediated activation of a specific subset of LTR-elements that are expressed during ZGA (Fig. 4A). Both HSV-1 infection as well as DUX4 overexpression leads to an induction of the MLT- and THE-class of retroelements, indicating that the expression upon herpesviral infection is driven by DUX4 (Fig. 4A). In contrast, only HSV-1 infection induced the LTR12C class of retroelements. There expression was independent of DUX4 (Fig. 4A), Of note, the previously described C10rf159 antisense transcript induced upon infection ^20^ starts at an LTR12C element within the first intron of its host gene and could thus be connected to retroelement activation. Moreover, a comparison of upregulated genes from HSV-1 infection, 8-cell stage of human development and FSHD patients showed that 843 host genes are significantly induced both during herpesviral infection and during the 8-cell stage of early human development (Fig. 4B) ^31^. This indicates that herpesviral induction of the germline-specific transcription factor DUX4 activates a transcriptional program of ZGA-specific genes and retroelements.

**Figure 5:**
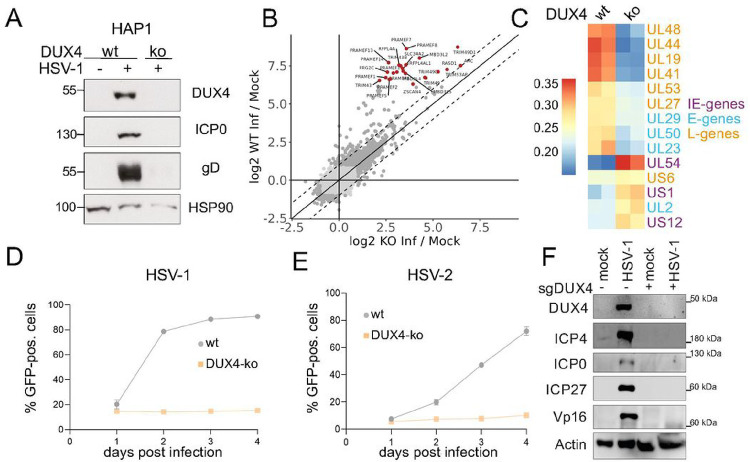
DUX4 expression is critical for HSV-1 and HSV-2 replication **A** DUX4 ko HAP1 cells were generated with CRISPR/Cas9 and DUX4 specific sgRNAs. HSV-1 protein expression in HAP1 wt cells and HAP1 cells with DUX4 knockout. Cells were infected with HSV-1-GFP (MOI 2) for 20h and analyzed by western blot. Representative experiment out of n=3. **B** mRNA expression (RNA-Seq.) of all host genes of HAP1-DUX4 ko cells plotted against HAP1 wt cells, both infected with HSV-1-GFP (MOI1) for 8h. Depicted in red are known DUX4 target genes. **C** Heatmap of mRNA-expression of selected viral transcripts from HSV-1 (MOI 1) infected wt and DUX4 ko HAP1 cells 8h post infection. Marked are viral genes with immediate-early, early and late expression kinetics. **D** GFP-expression of HAP1 wt and HAP1 DUX4-ko cells infected with HSV-1-GFP (MOI 0.1) at day 1–4 post infection, measured by flow cytometry. One representative experiment out of n=3. **E**GFP-expression of HAP1 wt and HAP1 DUX4-ko cells infected with HSV-2-GFP (MOI 0.05) at day 1–4 post infection, measured by flow cytometry. One representative experiment out of n=3. **F** Western blot of 293T WT cells and 293T DUX4 KO cells infected with HSV-1 for 18 h. Comparison of HSV-1 protein expression in WT cells and cells with complete DUX4 KO. Representative experiment out of n=4.

Our data revealed a robust expression of DUX4 during lytic replication of all human herpesvirus subfamilies. We thus wanted to address the physiological consequences concerning herpesviral gene expression and replication. DUX4 is located within the 4q35 region of chromosome 4 ^32,33^ and the gene is particularly complicated to target by CRISPR/Cas9. In healthy individuals the locus consists of 10–100 repeat units, and a copy of DUX4 resides in every unit. However, only one of the D4Z4 repeat arrays, the most distal one adjacent to the telomeres is encoding for the functional protein ^17^. In addition to multiple repeats on chromosomes 4 (4q allele) there is also another heterochromatic repeat array on chromosome 10 (10q allele) ^34^ which is likely to interfere with CRISPR/CAS9 based knockout strategies ^35^. We finally managed to generate knockout cells in the haploid cell line HAP1 (Fig. 5A). Upon infection of HAP1 DUX4 knockout (ko) cells, we could not detect a DUX4 specific band in the Western Blot, which is present in the infected wildtype (wt) HAP1 cells. Interestingly, the expression of the viral proteins ICP0 and glycoprotein D (gD) was also strongly reduced in DUX4 ko cells compared to wt cells. In order to assess the effect of DUX4 knockout on the transcription of the entire viral genome, RNA-seq. from HSV-1 infected knockout and wildtype cells 8h post infection was performed. Comparison of host gene expression from DUX4-wt and -ko cells showed that DUX4 target genes are significantly downregulated in the ko cells, whereas transcription of other genes is not altered (Fig. 5B). Unsupervised clustering showed altered expression of most HSV-1 genes in ko cells compared to wt cells (Fig. 5C, Fig. S7). Whereas the expression of early and late genes is higher in wildtype cells at 8h post infection, the expression of immediate-early genes like UL54 or US1 is lower in the wildtype and higher in the knockout cells, indicating that DUX4 is required for the later stages of HSV-1 infection (Fig. 5C). Infection experiments with GFP-expressing HSV-1 and HSV-2 showed that the infection does not proceed in DUX4 ko cells compared to wt cells where the virus replicate to normal levels (Fig. 5D,E). In order to confirm the results from our DUX4 ko HAP1 cells, we also used a transient approach in 293T cells to knockout DUX4 at the population level, which resulted in a complete knockout of DUX4 for a short time period. Infection of DUX4 ko 293T cells resulted in an almost complete loss of most HSV-1 genes tested in Western Blot, like ICP0, ICP4, ICP27 and VP16 compared to wt 293T cells (Fig. 5F). This confirmed the results obtained in the HAP1 cell line and shows that DUX4 is critical for HSV-1 and HSV-2 replication.

## Discussion

In humans the female oocyte is produced during female gametogenesis in the embryo and is then stored in prophase I of the meiosis for up to 50 years. Oocyte transcription is halted by epigenetic mechanisms, and stored mRNAs mostly control development^36^. After fertilization the zygote is formed, maternal to zygotic transition (MZT) takes place and with the onset of ZGA the zygotic genome starts to control transcription ^36^. In order to induce transcription, the embryo has to overcome silencing of the genome, which is regulated by repressive epigenetic modifications like DNA methylation, histone modifications and a shortage of the cellular transcription machinery ^36,37^. DUX4 has been shown to be important for ZGA by activating hundreds of genes that are necessary for further development, and several endogenous retroelements ^8–10,13^. Although the detailed function of most of DUX4 target genes remains elusive, it is thought that DUX4 induces several important factors that are involved in the creation of a permissive environment that allows transcription from the newly formed diploid genome.

Upon herpesviral infection the viral genome enters the nucleus and gets chromatinized, although the degree of chromatinization of the viral genome is discussed controversially, in particular for HSV-1. However, it is widely accepted in the field that herpesvirus genomes are subjected to epigenetic silencing, and that they evolved strategies to prevent epigenetic silencing of their genome in order to allow transcription necessary for viral replication and virus transmission ^38^. We show that herpesviruses from all human subfamilies as well as Papillomaviruses induce a robust expression of DUX4 upon lytic infection (Fig. 1A–E). Considering that alpha-, beta-, and gamma-herpesviruses were split into three separate lineages about 100–200 million years ago ^39^, this hints at a highly conserved mechanism in the coevolution of herpesviruses with their respective hosts. For HSV-1, we demonstrate that DUX4 expression is induced by viral tegument/immediate-early proteins ICP0 and ICP4, indicating that this is an active induction by herpesviruses (Fig. 2C). ICP4 directly binds to the DUX4 locus, however, for full DUX4 induction the E3-ligase activity of ICP0 is needed (Fig. 2C), indicating that ICP0 degrades a cellular protein that mediates silencing of the DUX4 locus. ICP0 and ICP4 induce expression of DUX4 at early stages of infection for a brief period, indicated by nuclear staining of DUX4 in ICP4+/VP26− cells (Figure 2F). In ICP4+/VP26+ double positive cells we either observed no DUX4 staining or cytoplasmic DUX4 staining (Figure 2F). Blocking viral DNA replication with PAA results in higher levels of DUX4 and its target genes, suggesting DUX4 behaves similarly to the strong HSV-1 beta genes, which are upregulated early in infection and are shut down during the switch to viral DNA replication and late gene expression (Figure 2D). Interestingly, this very short induction of DUX4 resembles the mechanism of DUX4 induction during ZGA, where a short burst of DUX4 is sufficient for target gene activation and reprogramming of the embryo but prevents toxicity mediated by DUX4.

Moreover, we could demonstrate that herpesviral DUX4 induction is essential for efficient herpesviral gene expression and replication. Depletion of DUX4 from cells results in a strong reduction of most herpesviral gene and protein expression, as shown for HSV-1 (Fig. 5A, C and F). In addition, we show that DUX4 can directly bind to the HSV-1 genome (Fig. 3A) and that HSV-1 as well as HSV-2 show drastically reduced viral replication in the absence of DUX4 (Fig. 5D and E). We hypothesize that herpesviruses evolved to partially mimic ZGA by actively inducing DUX4 expression. ZGA is conserved in all animals, and due to its significance in embryonic development there is very little room for the host to antagonize this viral mimicry of ZGA ^36^. Any interference with DUX4 function, for example by mutations in the coding sequence or by preventing DUX4 expression could lead to drastic changes in the embryonic development that are incompatible with life. Thus, from a viral perspective, it is beneficial to exploit a host gene which is essential for development for its own purpose in order to limit mutations that affect viral replication.

In addition to its role in ZGA and in the development of FSHD, it was published that DUX4 also plays an important role in a variety of human cancers ^40^. Reanalysis of almost 10.000 cancer transcriptomes from The Cancer Genome Atlas (TCGA) showed DUX4 re-expression in many human cancers ^40^. The authors speculate that expression of DUX4 and DUX4 target genes may contribute to tumorigenesis ^40^. Interestingly, the human gamma-herpesviruses, Epstein-Barr Virus (EBV) and KSHV are classified as human carcinogens by the WHO and cause cancer in humans^5^. We observe expression of DUX4 target genes in EBV-positive nasopharynx carcinoma cells, but not in healthy tissue from the same donors (Fig. 1G). The same holds true for patient cells of HPV-positive cancer (Fig. 1F). This indicates that the observed DUX4 induction is also of relevance *in vivo*. It is worth speculating but beyond the scope of this manuscript to investigate whether DUX4 expression induced by EBV, KSHV and HPVs also contributes to viral oncogenesis. In addition, Chew et al. showed that DUX4 expression results in a downregulation of MHC-Class I expression by interfering with STAT1 signaling, hinting at an immune evasion mechanism ^40,41^ that might also be important for viral infections.

We demonstrate that herpesviral DUX4 expression also leads to expression of several endogenous retroelements, including LTR-containing retrotransposons, LINE-1 elements and Alu-elements. Whereas cell type specific differences in the induction of retroelements exist, with more retroelements being transcribed in tumor cell lines than in primary cells, we identified an Alu-element 5’ of the ZSCAN4 gene that serves as a binding site for DUX4 and drives ZSCAN4 expression in all cell lines tested. In addition it is known for years that herpesviral infection leads to the induction of endogenous HERV-W retroviruses and also to the reactivation of the HIV-LTR, hinting at a role of herpesviral DUX4 in respective processes. It is hypothesized that some herpesviruses including HSV-1 have evolved a high GC-content in their genome in order to prevent insertion of endogenous retroelements that have a bias for AT-rich sequences. The active induction of DUX4 by HSV-1 proteins and the importance of DUX4 for herpesviral gene expression / replication supports this possible explanation for the high GC-content of HSV-1 genomes. It may help to preserve herpesviral genome integrity despite the DUX4-mediated induction of endogenous retroelements that could integrate into the viral genome.

Our data points at a very important if not essential role for DUX4 in herpesviral gene expression and replication. We could demonstrate that depletion of DUX4 in cells impairs viral replication. As such, it is tempting to speculate that DUX4 and DUX4 downstream genes could be targeted for therapy of herpesvirus-associated diseases. Blocking DUX4 means preventing herpesviral gene expression and subsequent viral replication from the beginning. Targeting a cellular protein has the major advantage that viral escape mutants and resistance-formation are unlikely, but usually this comes with the risk of possible side-effects. However, DUX4 is not expressed in adult somatic tissue, therefore side-effects should be negligible and DUX4 might serve as an attractive target for anti-herpesviral therapy.

## Methods

### Cell culture and viruses

HEK 293T, primary HFF and Vero cells were maintained in Dulbecco’s modified Eagle’s medium (DMEM, Thermo Fisher Scientific) supplemented with 10% (vol/vol) heat-inactivated fetal bovine serum (FBS, Thermo Fisher Scientific), 2 mM GlutaMAX (Thermo Fisher Scientific), 1 mM HEPES (Thermo Fisher Scientific) and 1% gentamycin (vol/vol) or penicillin-streptomycin (vol/vol). HAP1 were cultured in Iscove’s Modified Dulbecco’s Medium (IMDM,Gibco) supplemented with 10% (vol/vol) fetal bovine serum (FBS, Anprotec) and 1% (vol/vol) penicillin-streptomycin (Sigma-Aldrich). iSLK harbouring recombinant (r) KSHV.219 were cultured in DMEM supplemented with 10% (vol/vol) FBS, 2 mM GlutaMAX (Thermo Fisher Scientific), 10 mM HEPES buffer (Thermo Fisher Scientific) and 1% penicillin-streptomycin (vol/vol) , 1 μg/ml Puromycin and 250 μg/ml C418 . All cells were kept under standard culture conditions and monthly checked for mycoplasma contamination (MycoAlert Kit, Lonza).

Lytic replication of KSHV in iSLK.219 was induced by adding 1 μg/ml doxycycline. Infection experiments were performed with HSV-1 strain KOS, HSV-1 GFP (F-strain, provided by B. Kauffer, Berlin), HSV-2-GFP (strain MS, kindly provided by Beate Sodeik, Hannover) or HCMV (strain Ad-169). HSV-1 deltaICP0 (ICP4-YFP) and HSV-1 delta ICP27 were provided by R. Everett (University of Glasgow). HSV-1 T-VEC del ICP34.5/ICP47 IMLYGIC^®^, Talimogene laherparepvec) was provided by L. Heinzerling (Friedrich-Alexander-Universität Erlangen-Nürnberg). HSV-1 expressing ICP4-YFP and VP26-RFP is a new recombinant virus generated in the background of strain 17 by recombining HSV-1 expressing ICP4-YFP (obtained from Matthew D. Weitzman) and HSV-1 expressing VP26-RFP (obtained from Oren Kobiler) and plaque purifying for 5 cycles.

### Reagents, plasmids and transfections

Transfections were performed with GenJet (SignaGen Laboratories) or Lipofectamin 2000 (Thermo Fisher Scientific) according to the manufacturer’s protocol. Plasmids are listed in Table 3.

### Western Blotting

Cells were lysed in RIPA HS buffer (10 mM Tris-HCl pH 8.0, 1 mM EDTA, 500 mM NaCl, 1% Triton X-100 (vol/vol), 0,1% SDS (vol/vol), 0,1% deoxycholic acid (DOC) with Aprotinin and Leupeptin, MG-132 and sodium metavanadate (Sigma-Aldrich). The cell pellet was centrifuged at 4°C, 14.000 rpm for 30 min. Samples were diluted with Laemmli-SDS sample buffer and heated for 5 min at 95°C. Antibodies used are listed in Table 4.

### qRT-PCR

RNA was extracted using the Direct-zol RNA Miniprep Plus kit from Zymo Research according to manufacturer’s instructions. Reverse transcription was performed using the Super Script IV Kit (Thermo Fisher Scientific) according to manufacturer’s instructions. qRT-PCR was carried out using TaqMan^™^ Universal PCR Master Mix I (Applied Biosystems, Thermo Fisher Scientific) 0,1 μg as template on a 7500 Fast Real-Time PCR machine. Or reverse transcription and qRT-PCR were conducted in one step using 0.1 μg RNA as template with the Luna Universal Probe One-Step RT-qPCR kit (New England Biolabs) following manufacturer’s protocols. Primers/probes are listed in Table 1. Expression levels for each gene were obtained by normalizing values to *HPRT1* or *VTRNA* and fold induction was calculated using the comparative CT method (ΔΔCT method).

### CRISPR and sgRNAs

All sgRNAs used in this study were previously described and are listed in Table 2. sgRNAs were cloned into LentiCRISPRv2 plasmid gifted from F. Zhang (Addgene plasmid #52961) and verified by sequencing. Lentiviruses were packaged with pMD2.G (Addgene plasmid #12259) and psPAX2 (Addgene plasmid #12260) (both gifted from D. Trono) into HEK 293T. HEK 293T were seeded into 12 well plates and lentiviral supernatants added at 70–80% confluency. Plates were centrifuged at 1200 rpm for 2 min after centrifugation culture medium was added and cells incubated over night at 37°C. The medium was changed to normal culture medium the next day and selection with 2 μg/ml puromycin in normal culture medium started on day 3.

### DUX4 protein purification

The DUX4 protein was purified using the Intein Mediated Purification with an Affinity Chitin-binding Tag (IMPACT) system (NEW ENGLAND BioLabs INC). The coding sequence of DUX4 (Addgene: Plasmid #21156) was subcloned into the pTYB12 vector using EcoR1 and Sap1 restriction sites, with an intein-CBD tag added to the N-terminus of DUX4. Protein expression was induced by adding 0.4 mM of IPTG to ER2566 cells at an OD600 = 0.5 overnight at 18°C. Bacterial pellets were then resuspended in Lysis Buffer (20 mM Na-HEPES, 500 mM NaCl, 1 mM EDTA, 0.1% Triton X-100 and protease inhibitors (cOmplete^™^ Proteasehemmer-Cocktail)) and lysed using a French Press. The lysates were centrifuged at 15000 g at 4°C for 30 min, and the clarified lysate was slowly loaded onto the chitin column for purification using the ӒKTA pure^™^ chromatography system. The beads were then washed with 50 bed volumes of Column Buffer (20 mM Na-HEPES, 500 mM NaCl, 1 mM EDTA, and 0.1% Triton X-100) before protein cleavage with washing buffer containing 50 mM DTT. Finally, the eluate was further purified with Size Exclusion Chromatography using a Superdex 200 Increase 10/300 GL column (Cytiva).

### Next generation Chip-Seq

For CHIPmentation primary HFF cells were seeded in T175 flasks and infected with HSV-1 KOS (MOI 10) for different time points. CHIPmentation was conducted as previously described^44^. Cells were sonicated using the Bioruptor (Diagenode) for 30 cycles. For the immunoprecipitation protein G Dynabeads (Thermo Fisher Scientific) were used. Samples were incubated with either 2.5 μg Anti-DUX4 (E5–5) (Abcam) or Normal Rabbit IgG (Cell Signaling Technology) as control. Samples were purified using AMpureXP beads (Beckman Coulter) according to manufacturer’s description. Libraries were sequenced on HiSeq 4000 System (Illumina)

### Next generation RNA-Seq

HEK 293T wildtype cells and HEK 293T CRISPR/Cas knockout cells were seeded in T25 flasks. Cells infected for different time points with HSV-1 KOS (MOI 10). HAP1 and HAP1-DUX4 ko cells were Cells were lysed in Trizol (Life Technologies by Thermo Fisher Scientific), and total RNA was isolated using the RNA clean and concentrator kit (Zymo Research), according to the manufacturer’s instructions. Sequencing libraries were prepared using the NEBNext Ultra II Directional RNA Library Prep Kit for Illumina (NEB) with 9 cycles PCR amplification, and sequenced on a HiSeq 4000 device with 1×50 cycles. For quantification of viral gene expression alignments were done using hisat2^49^ on the HSV-1 genome (strain 17, genbank accession no. NC_001806) and readcounts per gene quantified using quasR^51^.

### CUT&Tag

Primary HFF cells were infected with HSV-1 GFP (MOI 1) in PBS supplemented with 0.1% Glucose and 1% FCS. the remaining virus was washed away with a low pH buffer (40mM Citric acid, 10mM KCl, 135mM NaCl, pH3) after one hour at 37°C. CUT&Tag was performed according to the manufacturing protocol (CUT&Tag-IT Assay Kit, Active Motif) using 2,5 μg Dux4 E5–5 (Abcam) or 2,5 μg rabbit IgG (Cell Signalling Technology). In short, cells were bound to concanavalin A-coated magnetic beads and permeabilized for subsequent incubation with primary and secondary antibodies. Specific cutting and addition of adapters was mediated by a protein A-Tn5 transposase fusion protein. Libraries were sequenced on HiSeq 4000 System (Illumina)

### EMSA

Viral DNA of HSV-1 GFP and HSV-1 KOS was isolated using the *Quick*-DNA MiniPrep kit from Zymo Research. Two short regions containing either the DUX4 binding motif or no DUX4 binding motif were amplified by PCR: about 600bp long fragments were amplified from HSV-1 genomes using the primers for Binding site 1 (Dux4 motif) BS1-fwd:gtgtaccactgctgtcg, BS1-rev:gtctgatcatgccccatacc, and Binding site 2 (No Dux4 motif) BS2-fwd:cgtgaaccaaagacgagggc, and BS2-rev:ccacgttgagaagctcgtcg. The EMSA was performed as described by Lee et al. ^45^ using 50ng of amplified viral DNA, which was incubated 30 min at room temperature with 1μg purified DUX4 protein and 2μL sperm DNA (D7656, Sigma-Aldrich).

### Flow cytometry

HAP1 wt and ko cells were simultaneously seeded and infected with HSV-1 GFP or HSV-2 GFP at a MOI of 0.1. At day 1, 2, 3 and 4 post infection, cells were detached by scraping, fixed in 1% PFA for 20 min and resuspended in FACS buffer (PBS supplemented with 2% FCS and 0.5mM EDTA). GFP expression was measured with BD LSRFortessa and the data was analyzed with FlowJo.

### Predicted DUX4 binding sites in different herpesviral genomes

The following viral genomes were downloaded at NCBI: HSV-1 (GU734771.1), HSV-2 (Z86099.2), HCMV (NC_006273), EBV (NC_007605) and KSHV (OK358814). The viral genomes were analysed for DUX4 binding sites with FIMO^46^ using the default parameters and the DUX4 binding motif obtained with CUT&Tag.

### Bioinformatic analysis

ChIP-seq data processing was done using the PiGx-ChIP-seq pipeline (https://doi.org/10.1093/gigascience/giy123). In short, adapters and low quality bases were trimmed from reads using Trim-galore. The reads were mapped on the hg19 version of the human genome, combined with HSV-1 genome, using Bowtie2 with k = 1 parameter. bigWig tracks were created by extending reads to 200, collapsing them into pileups, and normalizing to reads per million. Peak calling was done with MACS2 (https://github.com/taoliu/MACS) using the default parameters. Motif discovery was done using MEME ^47^ with the default parameters, on the top 100 peaks (sorted by q value), in a region of +/− 50bp around the peak center. Peak annotation was done using the hg 19 ENSEMBL GTF file, downloaded on 17.03.2017. from the ENSEMBL database ^48^. Peaks were annotated based on the following hierarchy of functional categories: tss -> exon -> intron -> intergenic (eg. if a peak overlapped multiple categories, it was annotated by the class that is highest in the hierarchy). Peaks were overlapped with the hg19 Repeatmasker repeat annotation, downloaded from the UCSC database on 03.02.2015.

For CUT&Tag analysis, the data was mapped to the hg19 version of the human genome with the viral genome using Bowtie2 with k = 1 parameter. bigWig tracks were created by extending reads to 200, collapsing them into pileups, and normalizing to reads per million. Peak calling was executed by MACS2 (https://github.com/taoliu/MACS) using the default parameters. The obtained peaks were filtered for being present in two consecutive time points and not present in the 2h time point. Motif discovery was done with MEME using the default parameters on all peaks of the human genome in a region +/−50bp around the peak center.

The bulk RNA-seq raw data of Human Adenovirus 5 infected cells (PRJEB57806), FSHD patients (GSE153301), and 8-cell-stage embryo cells (GSE36552), as well as the single-cell RNA-seq data of nasopharyngeal carcinoma patients (GSE162025), were downloaded from the NCBI database. The bulk RNA-seq data were aligned to the human genome (hg38) using STAR and then normalized using DESeq2. The R package VennDiagram was used to generate a Venn diagram showing the overlapping genes between the different samples. To visualize the DUX4 target genes expression patterns, the R package pheatmap was used to create a heatmap. For the single-cell RNA-seq data analysis of nasopharyngeal carcinoma patients, the STARsolo pipeline was used to align sequencing reads to the human reference genome hg38 and to generate feature-barcode matrices. The gene expression matrices for all PBMC and tumour cells were combined and converted to a Seurat object using the R package Seurat. The gene expression matrices were then log-normalized and linearly regressed using the NormalizeData and ScaleData function of the Seurat package. Finally, the scVirusScan pipeline was employed to identify viruses present in all PBMC and tumour cells.

### Transposon Quantification

Transposon expression was quantified using TeTranscripts ^49^ version 2.2.0, using the hg19 version of the human genome and a custom formatted transposon GTF file, made available by the Hammell laboratory from the following link: http://labshare.cshl.edu/shares/mhammelllab/www-data/TEtranscripts/TE_GTF/. Before visualization, the read counts per TF repName repeatmasker category were multiplied by the following size factors 10000/(number of repeats), and 1000/(average repeat length). The transposon expression was visualized relative to the uninfected wild type sample, using the ComplexHeatmap package (27207943).

### Comparison of HSV-1 gene expression with the embryonic expression profile

Expression profiles for HSV-1 and DUX4 ectopically expressed, 293T cells were taken from the following publication ^6^. The embryonic expression profiles were downloaded from the ARCHS4 database. The data originated from the following repository GSE44183. Data was visualized using the ComplexHeatmap function.

### Comparison of repeat expression during HSV-1 infection and DUX4 overexpression.

Expression profiles obtained from HSV-1 and DUX4 ectopically expressed, 293T cells were taken from the following publication ^6^. DUX4 binding data extracted from the supplementary data from the following publication ^14^. RNA - seq data was mapped using STAR, and the repeats were quantified by counting the number of, uniquely mapping, spliced reads overlapping with the each transposon category. Expression was visualized using ComplexHeatmap. Prior to the visualization the reads were normalized to uninfected samples.

### Statistical analysis

*P* values were calculated using an unpaired Student’s test. *P* <0.05 was considered statistically significant.

## Figures and Tables

**Table 1:** Oligonucleotides

Real time quantitative PCR probes		
DUX4L: 5’-TCAGCCAGAATTTCACGGAAG-3’ 5’-CCATTCTTTCCTGGGCATCC-3’	FAM/ZEN/3IABκFQ	IDT
ZSCAN4: 5’-CTAGTCACACATCAGCTCAGT-3’ 5’-TTCAGTCTCTTGCCTTGTGTC-3’	FAM/ZEN/3IABκFQ	IDT
TRIM49B: 5’-GCTGCTGAGGAACACCA-3’ 5’-ATTGCTTCTAGCCTTAAATTCACAT-3’	FAM/ZEN/3IABκFQ	IDT
TRIM48: 5’-ATCACTGGACTGAGGGACA-3’ 5’-GGGCGGATTTTGACGGT-3’	FAM/ZEN/3IABκFQ	IDT
ICP0: 5’-CGGACACGGAACTGTTCGA-3’ 5’-CGCCCCCGCAACTGC-3’	FAM/ZEN/3IABκFQ	IDT
HPRT1 : 5’-CGAGATGTCATGAAGGAGATGG-3’ 5’-TCAGCAAAGAACTTATAGCCCC-3’	HEX/ZEN/3IABκFQ	IDT
VTRNA1-1: 5’-TTTAATTGAAACAAGCAACCTGTCT-3’	FAM/NFQ	Thermo Fisher Scientific

**Table 2:** gRNAs

Small guide RNAs for CRISPR/Cas
sgRNA DUX4 #1 (E1–3; v1) 5’-CACCGCACCCGGGCAAAAGCCGGG-3’ 5’-AAACCCCGGCTTTTGCCCGGGTGC-3’
sgRNA DUX4 #2 (E1–4): 5’-CACCGCTGGAAGCACCCCTCAGCG-3’ 5’-AAACCGCTGAGGGGTGCTTCCAGC-3’
sgRNA DUX4 #3 (p8) 5’-CACCGTCGGACAGCACCCTCCCCG-3’ 5’-AAACCGGGGAGGGTGCTGTCCGAC-3’

**Table 3:** Antibodies

Primary Antibodies			
Anti-DUX4 (E5-5)	rabbit polyclonal	Abcam	Ab124699
Anti-DUX4 (clone 9A12)	mouse monoclonal	EMD Millipore	MABD116
VP16 (14-5)	mouse monoclonal	Santa Cruz Biotechnology Inc	Sc-7546
HSV-1 ICP0 antibody (11060)	mouse monoclonal	Santa Cruz Biotechnology Inc	Sc-53070
HSV-1/2 ICP27 antibody	mouse monoclonal	Santa Cruz Biotechnology Inc	Sc-69806
HSV-1 gD antibody	mouse monoclonal	Santa Cruz Biotechnology Inc	Sc-21719
HSV-1 ICP4 (H943) antibody	mouse monoclonal	Santa Cruz Biotechnology Inc	Sc-69809
KAP1 antibody	rabbit polyclonal	Bethyl Laboratories Inc	A300-274A
Purified Anti-Tif1β Phospho (S473) (Poly6446)	rabbit polyclonal	Biolegend	644602
TIF1β (H-300)	rabbit polyclonal	Santa Cruz Biotechnology Inc	Sc-33186
Purified Anti-ATM Phospho (Ser1981) antibody	rabbit polyclonal	Biolegend	10H11.E12
Phospho KAP1 (S824) antibody	rabbit polyclonal	Bethyl Laboratories Inc	A300-767A
Anti-HA.11	mouse monoclonal	Covance	MMS-101R
THE GFP antibody	rabbit polyclonal	Genescript	A01704-40
Anti-HCMV gB (27–287)	mouse monoclonal	Hybridoma	
Anti-ZSCAN4	rabbit polyclonal	Sigma-Aldrich/Merck Millipore	HPA006491
THE β-Actin antibody, HRP	mouse monoclonal	Genescript	A00730-40
GAPDH antibody, Biotin	goat	Genescript	A00915
B-Tubulin antibody	mouse monoclonal	Genescript	A01717-40

Secondary Antibodies		
Goat anti-mouse HRP	SouthernBiotech	1036-05
Swine anti-rabbit Immunoglobulins/HRP	Dako/Agilent	P039901-2
DyLight649 Streptavidin	Jackson	016-490-084
Donkey anti-Mouse IgG (H+L) Highly Cross-Adsorbed Secondary Antibody, Alexa Fluor 647	Invitrogen/Thermo Fisher Scientific	A31571
Goat anti-Rabbit IgG (H+L) Highly Cross-Adsorbed Secondary Antibody, Alexa Fluor 647	Invitrogen/Thermo Fisher Scientific	A21245
Donkey anti-Mouse IgG (H+L) Highly Cross-Adsorbed Secondary Antibody, Alexa Fluor 555	Invitrogen/Thermo Fisher Scientific	A31570
